# Longitudinal Analysis of the Premature Infant Intestinal Microbiome Prior to Necrotizing Enterocolitis: A Case-Control Study

**DOI:** 10.1371/journal.pone.0118632

**Published:** 2015-03-05

**Authors:** Yanjiao Zhou, Gururaj Shan, Erica Sodergren, George Weinstock, W. Allan Walker, Katherine E. Gregory

**Affiliations:** 1 The Genome Institute, Washington University, St. Louis, MO, United States of America; 2 Department of Pediatrics, Washington University School of Medicine, St. Louis, MO, United States of America; 3 Cooper Medical School of Rowan University, Camden, NJ, United States of America; 4 The Jackson Laboratory for Genomic Medicine, Farmington, CT, United States of America; 5 Department of Pediatrics, Mucosal Immunology and Biology Research Center, Massachusetts General Hospital for Children, Boston, MA, United States of America; 6 Department of Newborn Medicine, Brigham and Women’s Hospital, Boston, MA, United States of America; 7 Department of Nursing, Brigham and Women’s Hospital, Boston, MA, United States of America; 8 Harvard Medical School, Boston, MA, United States of America; College of Agricultural Sciences, UNITED STATES

## Abstract

Necrotizing enterocolitis (NEC) is an inflammatory disease of the newborn bowel, primarily affecting premature infants. Early intestinal colonization has been implicated in the pathogenesis of NEC. The objective of this prospective case-control study was to evaluate differences in the intestinal microbiota between infants who developed NEC and unaffected controls prior to disease onset. We conducted longitudinal analysis of the 16S rRNA genes of 312 samples obtained from 12 NEC cases and 26 age-matched controls with a median frequency of 7 samples per subject and median sampling interval of 3 days. We found that the microbiome undergoes dynamic development during the first two months of life with day of life being the major factor contributing to the colonization process. Depending on when the infant was diagnosed with NEC (i.e. early vs. late onset), the pattern of microbial progression was different for cases and controls. The difference in the microbiota was most overt in early onset NEC cases and controls. In proximity to NEC onset, the abundances of *Clostridium sensu stricto* from Clostridia class were significantly higher in early onset NEC subjects comparing to controls. In late onset NEC, *Escherichia/Shigella* among Gammaproteobacteria, showed an increasing pattern prior to disease onset, and was significantly higher in cases than controls six days before NEC onset. Cronobacter from Gammaproteobacteria was also significantly higher in late onset NEC cases than controls 1-3 days prior to NEC onset. Thus, the specific infectious agent associated with NEC may vary by the age of infant at disease onset. We found that intravenously administered antibiotics may have an impact on the microbial diversity present in fecal material. Longitudinal analysis at multiple time points was an important strategy utilized in this study, allowing us to appreciate the dynamics of the premature infant intestinal microbiome while approaching NEC at various points.

## Introduction

Premature infants are disproportionally at risk for morbidity and mortality as a result of organs that are immature and ill equipped for extrauterine life. These children are especially prone to inflammatory disease as a result of a poorly-regulated immune system and an inappropriate inflammatory response[1–5]. Necrotizing enterocolitis (NEC), an inflammatory disease of the immature bowel that has been associated with aberrant intestinal colonization, represents the most common cause of gastrointestinal (GI) morbidity and mortality among premature infants and is a major contributor to poor growth and neurodevelopmental outcomes[6,7]. In spite of decades of research, the prevalence of NEC among infants born between 500 and 1500 grams persists at approximately 7 percent[8], and according to the National Institutes of Child Health and sHuman Development Neonatal Network, may range from 4 to 19 percent in infants born prior to 28 weeks of gestation[9]. In short, developing an improved understanding of the etiology of NEC so that more effective prevention and treatment interventions may be developed is needed.

Prematurity and accompanying intestinal colonization are the only consistently identified risk factors for NEC[6]. Thus, when prematurity cannot be prevented, intestinal colonization is the major modifiable risk factor contributing to NEC. As a result, better understanding the pattern of intestinal colonization and community structure of the microbiome associated with NEC is an important area of study in the etiology of the disease. The research conducted to date on intestinal colonization and the microbiome aspects of NEC in premature infants using non-culture based techniques has resulted in inconsistent findings. Some studies have been unable to demonstrate clear differences among cases and controls[10,11], while others have shown that the community structure of the microbiome of the premature infant gut prior to and at the time of disease onset is unique[12–16].

When evaluating differences in the microbiome at specific points in time, NEC cases appear to first diverge from that of controls as early as three weeks prior to disease[12]. At two weeks prior to disease, NEC cases have been shown to have an increased proportion of Proteobacteria and a decreased proportion of Bacteriodetes when compared to controls[14]. At one week prior to disease, the microbiome of cases and controls has also appeared differently[14,16]; however, these differences do not persist to within 72 hours of disease onset, which may be explained by the greater degree of heterogeneity in the microbiome under conditions of disease when compared to the more stable and similar nature of the microbiome in control infants[16]. Finally, at the time of diagnosis, NEC infants have been characterized by an overall lower diversity index than the average premature infant[13].

While these studies have aided in our understanding of the disease, the prospective analyses of NEC cases and healthy controls have reported on small sample sizes or at single time points, not allowing us to see the more dynamic nature of the microbiome as the infant is both acquiring their intestinal microbiome and approaching disease. Thus, the objective of this prospective case-control study was to measure differences in the intestinal microbiome over time at several time points as measured in fecal material obtained from premature infants who developed NEC and their unaffected controls.

By analyzing the 16S rRNA genes of 312 samples including 12 NEC cases and 26 controls, we found that the microbiome undergoes dynamic development during the first two months of life for both infants with NEC and their healthy controls. We also found that the pattern of microbial colonization was different for cases and controls in proximity to disease onset. The difference in the microbiome was most overt in cases of early onset NEC (< = 22 days at diagnosis) and their controls.

## Methods

### Ethics Statement

Samples were collected following a protocol that was approved by the Partner’s Human Research Committee (IRB) for Brigham and Women’s Hospital. All study procedures were approved by the IRBs at both Brigham and Women’s Hospital in Boston, MA and at The Genome Institute in St. Louis, MO. The IRB deemed this study to be of minimal risk with no interaction and no intervention with human subjects and thus, was exempt from consent.

### Patients and samples

All infants were born at Brigham and Women’s Hospital in Boston, MA and cared for in a single-center Newborn Intensive Care Unit (NICU). Fecal samples were collected from preterm infants born prior to 32 weeks of gestation from birth to discharge or 60 days of life, whichever came first. We attempted to collect the stool samples in a daily basis, depending on when fecal samples were available. Briefly, diapers with fecal samples were collected by the bedside nurse, placed in a specimen bag, and stored at 4°C for no more than 24 hours. Samples were processed daily, which involved extraction of fecal material from infant diapers using sterile procedures, and immediately frozen at -80°C degrees until analyzed.

All infants were prospectively monitored for signs and symptoms of NEC. NEC was defined as the presence of clinical criteria fulfilling modified Bell’s Stage II or III NEC[17], and as confirmed by the clinical team caring for the infant. Infants who met criteria for Spontaneous Intestinal Perforation (SIP) were excluded from this study[18].

All case infants (n = 12) were paired with two gestational age and temporally matched controls (n = 26). Two control infants were part of a twin pair and therefore, in two cases, three infants served as controls to one NEC case, resulting in 26 total controls. In addition to selecting controls based on gestational age and temporal proximity to NEC cases, all controls were required to have multiple fecal samples that were collected at approximately (±2 days) the same day of life as the matched case, thereby accounting for the potential influence of day of life on the development of the microbiome. Comparison of demographics between case and control groups were performed based on the data type as listed in Table 1.

**Table 1:** Demographics of case and control groups.

	Case (n = 12)	Control (n = 26)	Statistical test	p-value	Adjusted p- value
Gestational age (GA) (weeks of completed gestation at birth)	27.8 (24–31)	27.9 (24–31)	t-test	0.84	1
Birth weight (BWT)	1048	1092	t-test	0.72	1
(grams)	(940–1860)	(520–1800)			
Sex (males)	7 (58%)	14 (54%)	Chi-square test	1	1
Mode of delivery			Fisher’s exact test	0.71	1
C-Section	9 (75%)	17 (65%)			
Vaginal Birth	3 (25%)	9 (35%)			
Race			Fisher’s exact test	0.003	0.04
White	3 (25%)	14 (54%)			
Black	6 (50%)	10 (38%)			
Other	3 (25%)	2 (8%)			
Apgar scores			Wilcoxon rank-sum test		1
1-minute	5 (1–8)	6 (1–9)		0.22	
5-minute	7 (4–9)	8 (2–9)		0.29	
Maternal infection prior to birth	3 (25%)	5 (19%)	Fisher’s exact test	0.69	1
Maternal antibiotic exposure prior to birth	4 (33%)	6 (23%)	Chi-square test	0.69	1
Infant sepsis(culture positive)	3 (25%)	7 (27%)	Fisher’s exact test	1	1
Antibiotic exposure days prior to NEC	9 (4–33)	4.5(0–29)	Wilcoxonrank-sum test	0.06	0.78
Feeding History			t-test		
Total enteral volume	1337	1963		0.09	1
fed prior to NEC	(5–3492)	(147–7165)			
Total enteral volume of breast milk fed prior to	831	1456		0.09	1
NEC	(03352)	(0.6020)			
NEC treatment		NA	NA	NA	NA
Medical	6 (50%)				
Surgical	6 (50%)				
Mortality associated with NEC	2 (17%)	NA	NA	NA	NA

Data are presented as means and ranges or number and percent. Maternal infections included chorioamnionitis. Infant sepsis was defined as any culture positive sepsis. Feeding history is described as the total enteral volume fed prior to time of NEC and the total enteral volume of breast milk fed prior to time of NEC. Antibiotic exposure and feeding history data on controls is reported for the time period prior to day of NEC diagnosis in the matched case.

GA, BWt, Apgars, Antibiotic exposure days, feeding history = mean and range

Sex, mode of delivery, maternal infection & antibiotics, infant sepsis = number (%)

### DNA Extraction, sequencing, and sequence data processing

All samples included in the analysis (n = 312) were transferred to barcoded specimen tubes, systematically placed in specimen boxes, packed in dry ice, and shipped to Washington University in St. Louis (WUSTL). These procedures prevented any samples from a thaw cycle during transit to WUSTL, where bacterial DNA was extracted from approximately 200 mg of fecal material following the QIAgen QIAamp DNA Stool Minikit Protocol and using the QIAcube (Qiagen, Germany). Isolated DNA was then transferred to The Genome Institute at WUSTL for sequencing and analysis.

The V3–V5 region of the 16S rRNA gene was amplified using primers 357F (5′- CCTACGGGAGGCAGCAG-3′) and 926R (5′- CCGTCAATTCMTTTRAGT-3′). Primers also contained an adaptor sequence and one of 96 tags unique to each sample. PCR was performed with the following conditions: 30 cycles of 95°C 2min; 50°C 0.5min and 72°C 5min. Amplicons were purified, pooled at equimolar concentrations, and pyrosequenced on the Roche 454 Titanium platform using the protocol developed by Human Microbiome Project[19].

During the process of library preparation and sequencing, negative controls are included in each pool and first checked by gel electrophoresis. Sequencing is conducted for negative controls that show no DNA contamination on the gel. This step ensures no strong contamination coming from the environment. After sequencing, the number of reads from negative controls is examined. If a large number of reads (>500 reads) is present in a negative control, the pool is discarded and re-sequenced.

Data processing and quality control (QC) was performed according to standardized protocols developed by the Human Microbiome Project[19]. In brief, samples were binned by allowing one mismatch in the barcodes. Reads were filtered to remove those samples with average quality score less than 35 and/or read length less than 200 nt. Chimeric sequences were removed using Chimera-Slayer [20]. Samples with less than 1000 reads were removed from the analysis. Samples passing QC were then classified from phylum to genus level using the Ribosomal Database Project (RDP) Naive Bayesian Classifier (version 2.5, training set 9). Taxa assigned with less than 0.5 confidence were reassigned to the next higher taxonomic level in which the classification threshold was greater than 0.5.

### Exploratory and multivariate testing of bacterial community structure and diversity

After initial sequence data processing, a taxonomical matrix was constructed with row as taxonomical classification and column as subjects. The taxonomical matrix was rarefied to the minimal number of reads in the matrix using the vegan package in R[21] before any further analysis. We then used Nonmetric Multi-Dimensional Scaling (NMDS) to explore the microbiome data structure based on dissimilarity measurement (Bray-Curtis dissimilarity) between two samples. NMDS is ordination technique. It aims to discover the data pattern in N dimensional spaces. For microbiome data, it allows the investigator to identify the subject relationships based on the bacterial compositions and abundances.

The different distribution of demographic factors in case and control groups were tested based on data type as indicated in Table 1, and the P-values from multiple comparisons were corrected using a Bonferroni approach. Permutational Multivariate Analysis of Variance (PERMANOVA) was used for formal statistical testing whether the bacterial community structure differed between different variables. The relative abundances of the taxa were averaged for the subjects with multiple time points when performing PERMANOVA. PERMANOVA partitions the Bray-Curtis dissimilarity matrix among sources of variation, and used a permutation test with pseudo-*F* ratios to obtain the p-values. To extract the genera that contribute to the difference between two bacterial communities, we performed Metastats analysis[22]. Metastats is a statistical method based on Fisher’s exact test developed for the HMP study. The genera were considered to be significantly different if (1) adjusted P value <0.05 using false discovery rate approach, and (2) the mean relative abundance for a given genus was at least 1% in one group.

Univariables including richness and Shannon diversity were used to describe the bacterial complexity in a sample as previously reported[23]. Richness is defined as the number of different bacteria in a sample. Richness does not take the taxa abundances into account. Shannon diversity quantifies both bacteria composition and their abundance, and it is a popular diversity index used in ecology and human microbiome studies[23]. The diversity variation over time was modeled by mixed linear regression using lmer4 in R [24]. Day of life, gender, and mode of delivery were entered into the model as fixed effects. Subjects in the model were used as random effects. *P*-values were obtained by likelihood ratio tests of the full model against the model without the effect.

## Results

### Microbiome community structure in NEC cases and controls

We sequenced 312 stool samples from 38 individual subjects including 12 NEC cases and 26 controls with a median frequency of 7 samples per subject (IQR: 5–10) and minimal 1 sample per subject. The median time interval of sample collection was 3 days (IQR: 1.3–5). The sample distribution in this dataset as a function of day of life was summarized in S1 Fig. The median day of life of NEC onset for the 12 NEC subjects was 25.5 (IQR: 16.8–37.0). The significant differences in the clinical characteristics of the cases and controls pertained to race and the number of days of antibiotics infants received prior to the time of NEC diagnosis (before P value adjustment) (Table 1).

In total, 2.6 million high quality and chimera free reads were produced. Fifteen phyla and 333 genera were detected in this data set, which included infants from birth to 60 days of age. In general, zero to 50 reads are present in our negative controls. Because our study focused on the relatively high abundant organisms as described in the materials and methods, and because we also rarefied the reads down to 1000 reads per sample, the reads in the negative controls will have little impact on the results we have reported and the conclusion we have drawn.

Among all the taxa, Proteobacteria, Firmicutes and Actinobacteria were the three most abundant phyla, accounting for 57.4%, 41.2% and 1.0% of the total reads. *Clostridium sensu stricto*, representative of a genus in cluster I in Clostridium phylogeny[25], was the most abundant genus in this dataset. It was present in 64.4% of the samples, and accounted for 21% of the total reads in this dataset. The most prevalent genus was *Enterobacter*, present in 95% of the samples, indicating that this bacterium is likely to represent the longitudinal core genera during the early days of life (0–60 days) in premature infants. Other highly prevalent genera were *Staphylococcus* and *Escherichia/Shigella*, were also present in more than 80% of the samples.

**Fig 1:**
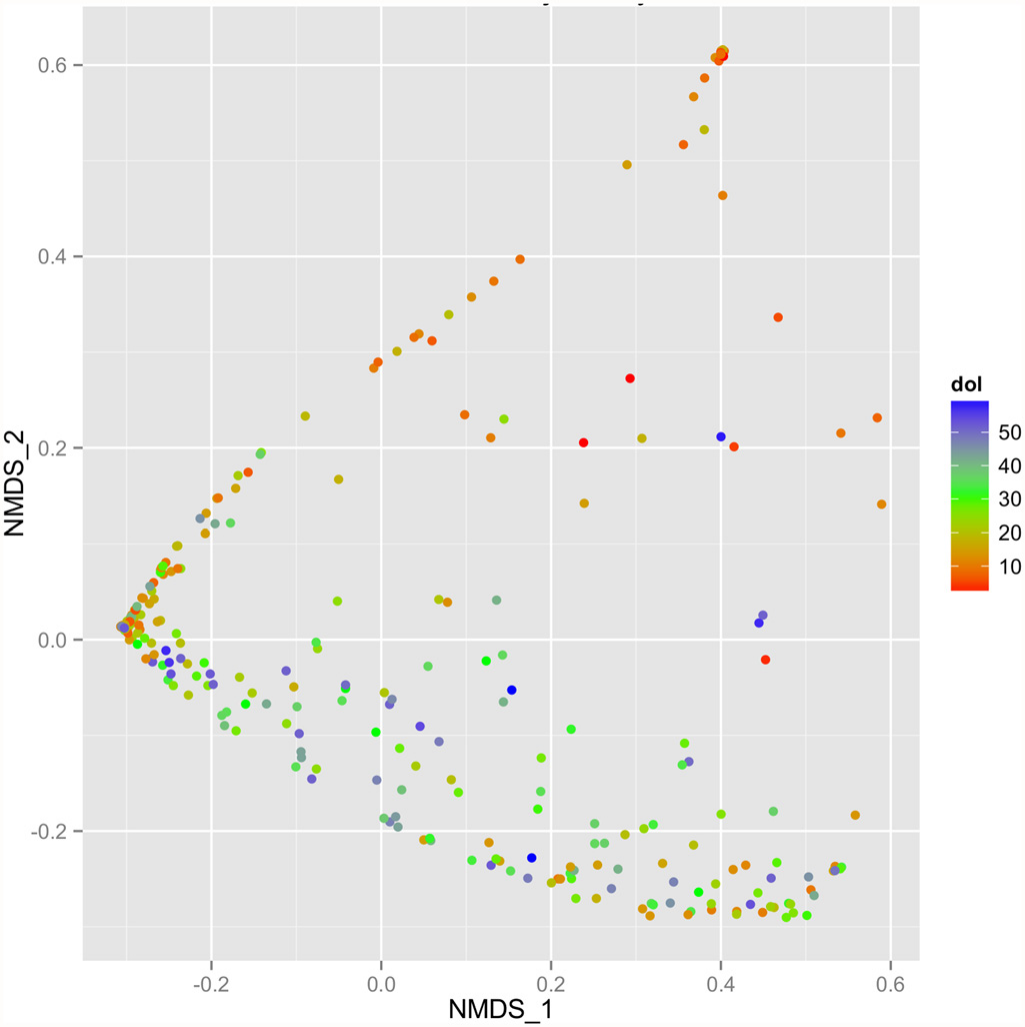
Premature gut microbiota variation in the first two month of life. The gut microbiota variation at class level over 0–60 days of life from all the samples is visualized by NMDS plot. The color gradient from red to blue represents the day of life of the babies. The microbiota from early day of life is distinguished from the elder days.

During early life, when the intestinal microbiome is first established, the microbes populating the gut are undergoing dynamic succession and multiple factors potentially contribute to this developmental process. To understand this developmental process, we examined how different factors affect the bacterial community structure. Bacterial community structure refers to the bacterial compositions and abundances in one sample or a group of samples. As shown in the NMDS plot, the day of life is the most striking driver of the development of the gut microbiota among these premature infants(Fig. 1, S2 Fig.), and this age dependent cluster pattern was especially evident in the samples analyzed at the genus level. Samples were not clustered by antibiotic usages and health status (NEC and healthy control) (S3 Fig.).

To examine the difference in the bacterial community between NEC and healthy controls at the day of NEC diagnosis, we illustrated the differences in the bacterial community of NEC subjects and their controls by NMDS plot. Six of the twelve NEC cases in this study had samples available for analysis at the time of disease onset. No clear separation was observed between NEC cases and the controls (S4A, C Fig.); PERMANOVA testing showed no statistically significant differences between NEC and controls at day of NEC onset (p = 0.52 for genus level and p = 0.40 at class level). Instead, at the genus level, the six NEC samples were segregated in the first dimension of the NMDS plot, with the three NEC samples from onset day 23 to 35 on the left side and the three NEC samples from onset day of life (DOL) 10–22 on the right side of the first dimension (S4C Fig.). Barplots of the top five abundant classes demonstrated that three NEC subjects (1, 2 and 3 DOL< = 22) were dominated by Clostridia with the relative abundances of 50.0%, 94.8% and 29.7%, respectively, while the other three NEC(4, 5,6) were dominated by Gammaproteobacteria with the relative abundances of 73.4%, 98.8% and 46.2%, respectively(S4B Fig.). At the genus level, three (NEC 1, 2 and 3 DOL< = 22) out of six NEC subjects were dominated by *Clostridium sensu stricto* from Clostridia class, while the NEC samples 4,5,6 with high abundance Gammaproteobacteria class were heterogeneous, with *Pseudomonas*, *Pasteurella*, *Serratia* and *Klebsiella* being the most prevalent genera, respectively (S4D Fig.).

The finding that the microbial community on the day of NEC onset did not cluster by disease is perhaps not surprising given the range in the day of life that NEC was diagnosed among these cases (day 10–35) and the strong influence of day of life on the gut microbiota as indicated above. To minimize the effect of day of life on the microbiome when comparing the NEC cases and their controls, we separated the NEC subjects into two groups: (1) early onset disease (day of NEC onset < = 22) and (2) late onset disease (day of NEC onset >22). In the absence of any consensus regarding cutoffs for early versus late onset NEC, we chose this interval after inspecting pilot NMDS data from samples obtained on the day NEC was diagnosed (S4C Fig.), as described above.

**Fig 2:**
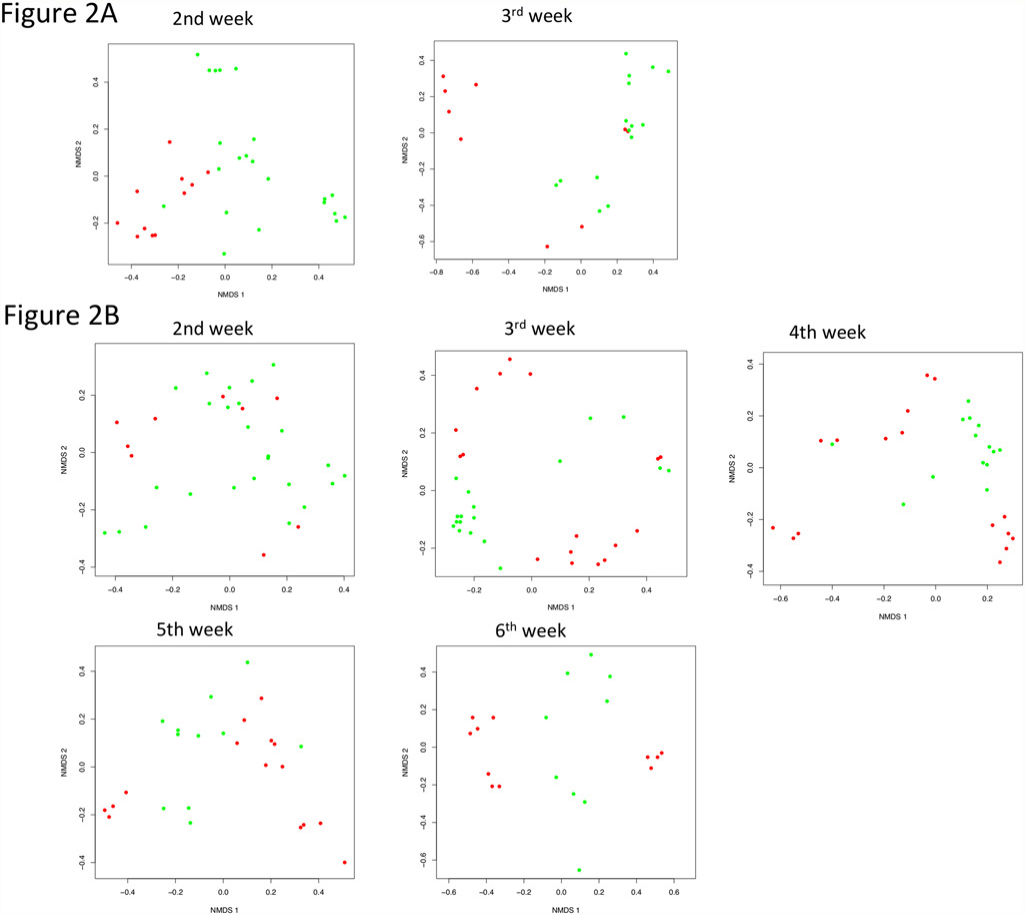
NMDS of early and late onset NEC and controls at the genus level. The difference between NEC and controls is displayed for early onset NEC (Fig. 2A) and late onset NEC (Fig. 2B) with their controls by NMDS plot. Each dot represents one sample. Green dots represent the controls and red dots represent the NEC samples. Early NEC subjects and control subjects have a clear separation at second week of the life. The distinction between late onset NEC and controls is less obvious except at the third week of life.

Based on the data available, the disease course was analyzed and NMDS was performed at the second and third week of life for the early onset NEC group, and from the second to sixth week of life for the late onset group. In these analyses, only data prior to or on the day of disease were included, ensuring that the findings at these time points represent the microbiome prior to the time of NEC diagnosis. Interestingly, we observed a clear separation of early onset NEC and controls at the second week of life at the genus levels (p = 0.01) (Fig. 2A, S1 Table), suggesting that the microbiota were different between early onset NEC samples and control. However, no significant difference was found between early onset NEC and controls at the third week of life (p = 0.36).

In NEC subjects, at the class level the median relative abundance of *Clostridia* was 56.4% at second week of life, but decreased to 29.7 at third weeks of life (S5B Fig., p = 0.02). However, in controls, *Clostridia* increased from 0.1% to 3.8% over the same time points. The median relative abundances of Gammaproteobacteria were 21.9 and 92.0% in NEC and control groups at second week of life, but then showed different trend patterns as they reached approximately 63.0% for both NEC and control at third week of life. At the same time points, the median relative abundance of Negativicutes showed minor changes in NEC and controls with no statistical significance (S5B Fig.). At the genus level, *Clostridium sensu stricto* was seven times more abundant in NEC cases than in controls at second week of life, and decreased significantly after one week in NEC (p = 0.05) but increased in controls although no significant difference was found (p = 0.22). *Pasteurella* was the only high abundant genus (22.7%) that increased in NEC samples at second week of life (p = 0.007). *Pasteurella* was not detected in controls (S5C Fig.).

In the late onset NEC group, the separation among cases and controls is not as evident as the early onset NEC cases and their controls (Fig. 2B), with only the first three weeks showing marginal significance (p = 0.06, p = 0.05, p = 0.05, respectively) at genus level (S1 Table). The NEC subjects were also distinct from each other, and the individualized distribution became more notable with increasing age, especially at six weeks of life. The latter observation was in line with the NMDS portrayal at day of NEC (S4 Fig.). While we have reported significant p values in all of the above analysis, these should be interpreted with caution given the small sample sizes included in both the early and late onset analyses.

### Progression of microbiota prior to NEC onset

**Fig 3:**
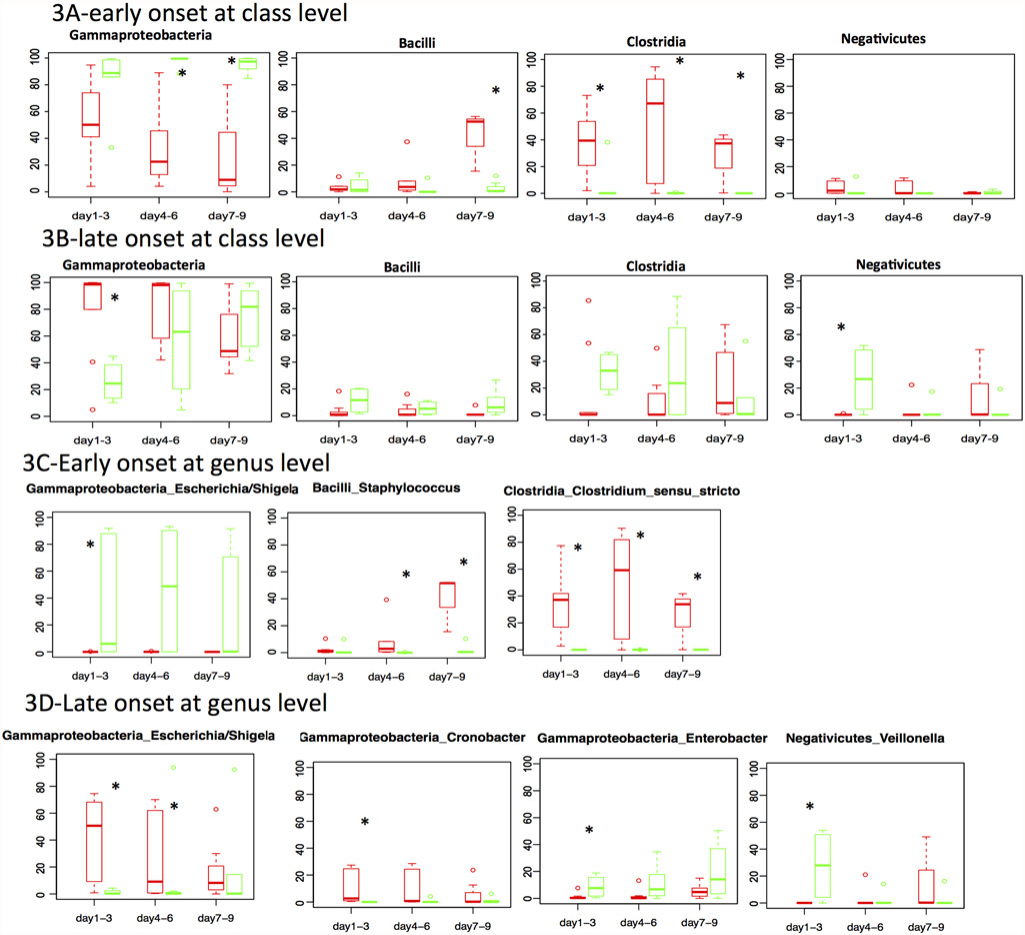
Microbiota progression before early onset NEC and late onset NEC at class and genus level. The relative abundances of four most dominant classes (Fig. 3A-early onset at class level, 3B-late onset at class level) and the genera (Fig. 3C-early onset at genus level, 3D-late onset at genus level) that are significantly different between NEC and controls are plotted at 7–9 days, 4–6 days and 1–3 days prior to NEC onset. In early onset NEC category, 3,6, 8 NEC samples were included at 7–9 days, 4–6 days and 1–3 days; 8, 5, 6 control samples were included in the above time points. In late onset NEC category, 7, 7,10 NEC samples and 6, 8, 4 control samples were included in the above time points. Red and green of the boxplots indicate NEC and control samples, respectively. The asterisks indicate the significant difference between NEC and control.

To characterize the differences in intestinal colonization between NEC cases and controls prior to disease onset, we compared the community structure of the microbiome in cases and controls from 7–9 days, 4–6 days, and 1–3 days prior to early onset and late onset disease. In analyzing the data in this manner, we found that prior to early onset NEC, four classes of bacteria showed interesting patterns in the microbiome 9 days prior to disease (Fig. 3A): (1) The amount of Gammaproteobacteria in controls maintained the same predominance for 9 days before NEC onset, yet it is significantly higher in controls than in NEC samples at 7–9 and 4–6 days prior to NEC onset (p = 0.01 and 0.008, respectively), and not significantly different just before disease onset as a result of Gammaproteobacteria from NEC cases showing an increasing trend while approaching disease. As seen in Fig. 3A, 1–3 days before NEC onset, Gammaproteobacteria in cases reached a similar level as the controls. *Klebsiella* is the major genus from Gammaproteobacteria in the early onset group, however, its relative abundance was not significantly different between NEC and controls. *Escherichia/Shigella was significantly higher in controls than early NEC cases* (p = 0.005) (2) Strikingly, bacteria from the Clostridia class (*Clostridium sensu stricto* as the major genus) in the NEC samples was found to be significantly higher than in the controls consistently from 1–9 days before NEC onset (p = 0.01,0.05 and 0.01 for 7–9, 4–6, 1–3 days before onset) (3) Bacilli (especially *Staphylococcus* genus) decreased within the 9 days of disease in the early onset NEC cases, while no obvious changes were detected in controls samples. (4) Negativicutes were low in abundance and we did not find any significant changes for bacteria in this class among the early onset group.

In analyzing the late onset group, we also found four interesting trends in the microbiome of premature infants with NEC as compared to their controls. (1) Gammaproteobacteria showed a steady decreasing trend in controls and increasing trend in NEC samples (Fig. 3B). The Gammaproteobacteria became significantly higher 1–3 days prior to the late onset NEC (p = 0.03). Genus level analysis demonstrated that not all of the genera from Gammaproteobacteria showed an increasing trend in NEC samples. *Escherichia/Shigella* indicated a drastic increase, as they became significantly higher than controls 1–3 days and 4–6 days prior to NEC (p = 0.02). *Klebsiella* and *Enterobacter* decreased towards diseases onset and *Enterobacter* became significantly lower in the 1–3 days prior to NEC. (2) The abundance of Bacilli (mainly *Enterococcus*) showed no significant difference between cases and controls, and their abundance did not show significant variation over the 9 days prior to late onset NEC (p>0.05). (3) In contrast to early onset NEC, *Clostridia* (mainly *Clostridium sensu stricto*) decreased or maintained a low abundance at the class level in samples representing NEC cases, while the control subjects demonstrated a microbial trajectory characterized by an increasing proportion of Clostridia within 9 days before disease. The abundances of Clostridia were not significantly different between case and controls at the three time points (p>0.05) (4) Negativicutes increased in the late onset control subjects while no obvious change was detected in the late onset NEC subjects. However, at the genus level, we found *Veillonella* was significantly higher in controls than NEC cases 1–3 days prior to NEC onset (p = 0.005).

### Microbial diversity in NEC cases and their controls

Previous prospective case-control studies have reported inconsistent findings on the microbial diversity associated NEC [10,12,14–16]. We used mixed linear regression to assess the effect of different variables including, day of life, mode of delivery, gestational age, and gender, on the microbial complexity among samples in this study. The microbial complexity measured by richness and Shannon diversity describes the number of different taxa and their abundances in a sample, respectively. The goal of using mixed linear regression on the microbial diversity is to evaluate the difference in diversity variation between comparison groups over time. In this study, we analyzed the diversity at the genus level, given the more complex bacterial community at a lower taxonomical level. Richness and Shannon diversity from the samples before NEC onset increased significantly over the 2 months of life (p = 9.76E-14 for Shannon diversity, and p = 0.003 for richness), indicating day of life is a critical factor affecting the complexity of the bacterial community.

The control samples have significantly higher richness (p = 0.03) but not Shannon diversity overtime, suggesting that the pace of colonization in NEC subjects is slower than their matched controls. Mode of delivery, gestational age, and gender showed no significant influence on the diversity measured (p>0.05).

In this analysis, antibiotic usage was not included in the NEC model because of the complexity in determining the influence of this variable on the outcome of the microbiome at a specific point in time. For example, the type, dosage, and duration of antibiotic usage may all have an effect on the intestinal microbiota. In this study, the infants received broad spectrum intravenously (IV) administered antibiotics (not oral antibiotics) following birth, which is a routine practice common to preterm infants in the NICU. While recent findings have shown that there is a disturbance in the gut microbiota associted with IV antibiotic therapy in adults[26], the molecular mechanisms require further study and whether or not this same influence is observed in preterm infants early in life is unknown.. Here, we performed an initial investigation relative to this issue by comparing the microbial diversity between healthy subjects who received antibiotics within 5 days of sample collection and subjects who did not have antibiotic usage at least five days before sample collection. We chose only the control subjects to exclude the potential effect of disease itself on diversity. The selected samples were obtained between days of life 7 to 14. The comparison was done after adjusting day of life, gender, feeding, mode of delivery and presence of maternal infection. The diversity including richness and Shannon diversity were lower in subjects treated by antibiotics within five days vs subjects who did not (S6 Fig.), with a marginally significant difference on richness (p = 0.09 for richness and 0.18 for Shannon diversity), suggesting IV antibiotic treatment may influence the complexity of the premature infant intestinal microbiome. As described above, NEC samples tended to have lower microbial diversity than controls, and they also have marginally more antibiotics usage than controls (Table 1 before p value correction). We speculate that the low diversity in NEC samples may be due in part to the increased exposure to antibiotics outside the treatment for NEC among these infants.

## Discussion

In our study, which consists of a larger cohort of premature infants and a greater number of samples at more frequent time points prior to disease than have been reported in other studies, we identified specific patterns of altered microbial composition prior to NEC that are unique based on when NEC was diagnosed in the infant’s life. The time dependent variation in the microbiome is not surprising because the infants’ intestinal microbiota is under rapid development in early life and the microbiome has been shown to be highly dynamic until approximately two years of age [27]. As seen in our results, the changing microbiota was largely affected by day of life and less by other clinical variables, which suggests that the microbiome comparison between NEC cases and their controls should be confined to a similar time frame following birth.

The day of NEC onset in our study spanned from day 9 to 55. This wide range in age at the time of disease is a confounder in the microbiome comparison between NEC cases and controls. This is evident from our NMDS plot using all of the NEC onset data in which early and late onset NEC samples were distinct. To deal with the influence of day of life on the microbiome community structure, we divided the NEC samples into early and late onset groups and compared the colonization pattern of the microbiome with their matching controls. We observed a clear separation of early onset NEC samples with their controls. However, this separation is less clear for late onset NEC samples and controls. In the latter case, the microbiome appears to have a more individualized pattern. The high inter-subject variation in late onset NEC and their controls is consistent with previous study on the process of intestinal bacterial colonization early in life [28, 29]. Previous studies showed NEC cases and controls were segregated, suggesting their microbiomes were different [12,14,16]. However, these studies are based on single sampling points or smaller sample size, considering the high inter-subject variation, future studies that involve larger sample sizes and cross-center data comparisons will have the ability to further explore the inter-subject variation that has currently been identified with greater accuracy.

Is there a common pathogen attributable to NEC? The pathogen that has been most commonly associated with NEC, Cronobacter sakazakii, has demonstrated that virulence is not a property of a bacterial species as a whole [30–33]. Rather, pathogenicity may be a function of the host response to the characteristics of specific strains within a certain species of taxa [30] In addition to Cronobacter, earlier studies using culture based techniques and recent studies using metagenomic approaches have linked several bacteria to NEC. These bacteria have primarily been identified as Gram-negative species (i.e. *Klebsiella pneumoniae*) [10,12,14,15,34,35]. However, there have also been observations suggesting that Gram-positive bacteria (i.e. *Enterococcus faecalis*) [36] and anaerobic species (i.e. Clostridium) may contribute to the etiology of NEC[37]. Our study investigating NEC using a metagenomic approach demonstrated an imbalanced bacterial community in NEC. By analyzing the data 9 days prior to NEC onset, we found a higher abundance of *Clostridium sensu stricto* (genus) from Clostridia (class) in early onset NEC samples when compared to their controls. This observation did not hold true in late onset NEC cases, where *Escherichia/Shigella* were higher. The inconsistent finding between early and late onset NEC may have resulted from the age difference of the infants at the time of disease, as the bacteria associated with NEC are influenced by the natural colonization process over time in these infants. Taken together, these findings suggest that a common specific pathogen associated with all NEC is lacking. This is not surprising, given that in the molecular and high throughput sequencing era of microbiology, where multiple PCR and metagenomic sequencing of biological specimens have the potential to discover numerous organisms or pathogens, the concept of one pathogen resulting in one disease is currently being challenged. Experimental animal model based validation is warranted to identify the real causative agents.

Longitudinal analysis at multiple time points has allowed us to recognize the dynamics of the microbiome while approaching disease. Focusing on the early onset NEC samples, we can see that it is not an absolute change in abundance of a specific bacteria that is most relevant when studying the microbiome in proximity to disease. Rather, it is the trend among the members of the microbiome over time that seems to be associated with disposition for disease. The abundance of Gammaproteobacteria in early onset NEC provides an important example of trending microbiome data. As seen in Fig. 3A, if we evaluate the differences in Gammaproteobacteria at discrete time points prior to NEC, we would likely conclude that Gammaproteobacteria is overwhelming protective against NEC (i.e. higher Gammaproteobacteria observed in controls at all of the time intervals before disease). However, the trend data as observed over the nine days preceding NEC shows that while NEC cases do indeed have a lower abundance of Gammaproteobacteria than controls, this abundance dramatically changes as the infant approaches disease. Therefore, NEC may evolve as a result of an increasing abundance of Gammaproteobacteria as the infant approaches disease. This changing trend is in stark contrast to the relatively unchanging abundance observed in controls at the same time points, suggesting that the change may be contributing as much to the etiology of the disease as the community structure of the microbiome.

While the overall community structure and balance between pathogenic and commensal bacteria within the intestinal microbiome is very likely to influence the development of NEC, to what extent the microbiome drives the etiology of NEC is not clear. As in many other diseases, where the disease evolves as a result of the interaction between the pathogen and the host, we think that there is a greater need to understand the premature infant inflammatory response to intestinal bacteria, as this response is likely playing a significant role in the etiology of NEC. This area of inquiry is especially important given recent findings, which have shown that the immune system of the infant born at term is “turned-down” for a period of time early in life, presumably to allow for the rapid intestinal colonization that occurs following birth [38]. How the immature immune system is programmed to facilitate early intestinal colonization of the premature infant is unknown. Future research on the immature immune response, in tandem with early intestinal colonization, may help develop interventions that attenuate the inappropriate inflammatory response that characterizes NEC in premature infants.

Future work on the preterm infant intestinal microbiome and NEC will require an in depth exploration of the influence of clinical variables such as mode of birth, antibiotic exposures, and nutrition [39]. Of these, nutrition is likely to be a significant contributor to the development of the microbiome and its role in the pathogenesis of diseases such as NEC. For example, studies have shown a protective effect of breast milk against NEC [40] due to the bifidogenic nature of breast milk. In addition, studies have shown that the intestinal microbiome is strongly influenced by diet during infancy [29,41,42]. Further analysis of the influence of nutrition on the microbiome and in association with NEC was outside the scope of the current study design as the infants included in this study were fed a mix of human milk and infant formula. Future study designs that have the ability to investigate groups of infants who are fed a diet that is exclusive for human milk or infant formula will be best positioned to explore the influence of nutritional exposures on the acquisition and development of the preterm infant intestinal microbiome.

In summary, there are discernible and potentially meaningful trends in the microbiome over time in premature infants who developed NEC. While this is the largest such study reported to date, its major limitation remains a small sample size. Fluctuations in microbial content in this interval in life, and the nearly 7 week interval of life over which NEC occurred, further reduce our ability to compare cases and controls. Therefore, we recommend that caution should be taken in the calculation and interpretation of the statistics applied to the study. Future studies with larger sample sizes are needed to more fully describe the differences in the premature infant intestinal microbiome associated with NEC.

## Supporting Information

S1 Fig Sample frequency and time Interval of sampling. X-axis represents individual subject. Y-axis represents the day of life that a given sample is collected.
(TIF)

S2 Fig Premature gut microbiota variation in the first two month of life at the genus level. The gut microbiota variation at genus level over 0–60 days of life from all the samples is visualized by NMDS plot. The color gradient from red to blue represents the day of life of the babies.
(TIF)

S3 Fig Microbiota from all the samples are not separated by case and controls as well as by the antibiotic usage. (A) NMDS plot at class level. Samples are colored by NEC or control groups (B) NMDS plot at genus level. Samples are colored by NEC or control groups (C) NMDS plot at class level. Samples are colored by the accumulated antibiotic usages. We assigned 1 for antibiotic usage and 0 without antibiotic usage. If multiple antibiotics were used at a given day, we summed up antibiotics to indicate the total antibiotic usage for the day. The color gradient shows the accumulated antibiotic usages overtime.
(TIF)

S4 Fig No difference between NEC and control at the day of NEC. (A) NMDS plot of microbiota in NEC and controls at the class level at the day of NEC (B) The relative abundances of top five microbiota in 6 NEC samples (top) and their corresponding control samples (bottom) at class level. The NEC subjects are labeled as 1–6. 1–3: the early onset subjects; 4–6: the late onset subjects. Their corresponding control samples have the same label as the NEC cases. (C) NMDS plot of microbiota in NEC and controls at the genus level at the day of NEC diagnosis (D) The relative abundances of top 25 microbiota in 6 NEC samples (top) and their corresponding control samples (bottom) at genus level. The samples are labeled exactly as (B). The color scheme for each of the five taxonomical classes in S4B Fig. matches at least one of the color for the taxa within the same class.
(TIF)

S5 Fig Microbiota comparison between early NEC and controls at the class level. Each dot represents one sample.. All the control samples are coded by green, and all the NEC samples were colored with red in the NMDS plots (A1 and A2). The relative abundances of the four bacterial classes at the second and third week of life for NEC and controls samples (B). The relative abundances of two bacteria genera that are significantly different between second and third week (C).
(TIF)

S6 Fig Low diversity in antibiotic usage control samples. Richness (A) and Shannon diversity (B) are shown for samples treated with antibiotics within five days and samples without antibiotics for at least five days. The samples are from NEC control group from second week of the life.
(TIF)

S1 Table PERMANOVA analysis of the early onset NEC and controls. (DOCX)
