# Supplementary figures and images for: Longitudinal Analysis of the Premature Infant Intestinal Microbiome Prior to Necrotizing Enterocolitis: A Case-Control Study
Source: PLoS One. 2015 Mar 5;10(3):e0118632. doi: 10.1371/journal.pone.0118632 (PMC4351051; doi:10.1371/journal.pone.0118632)

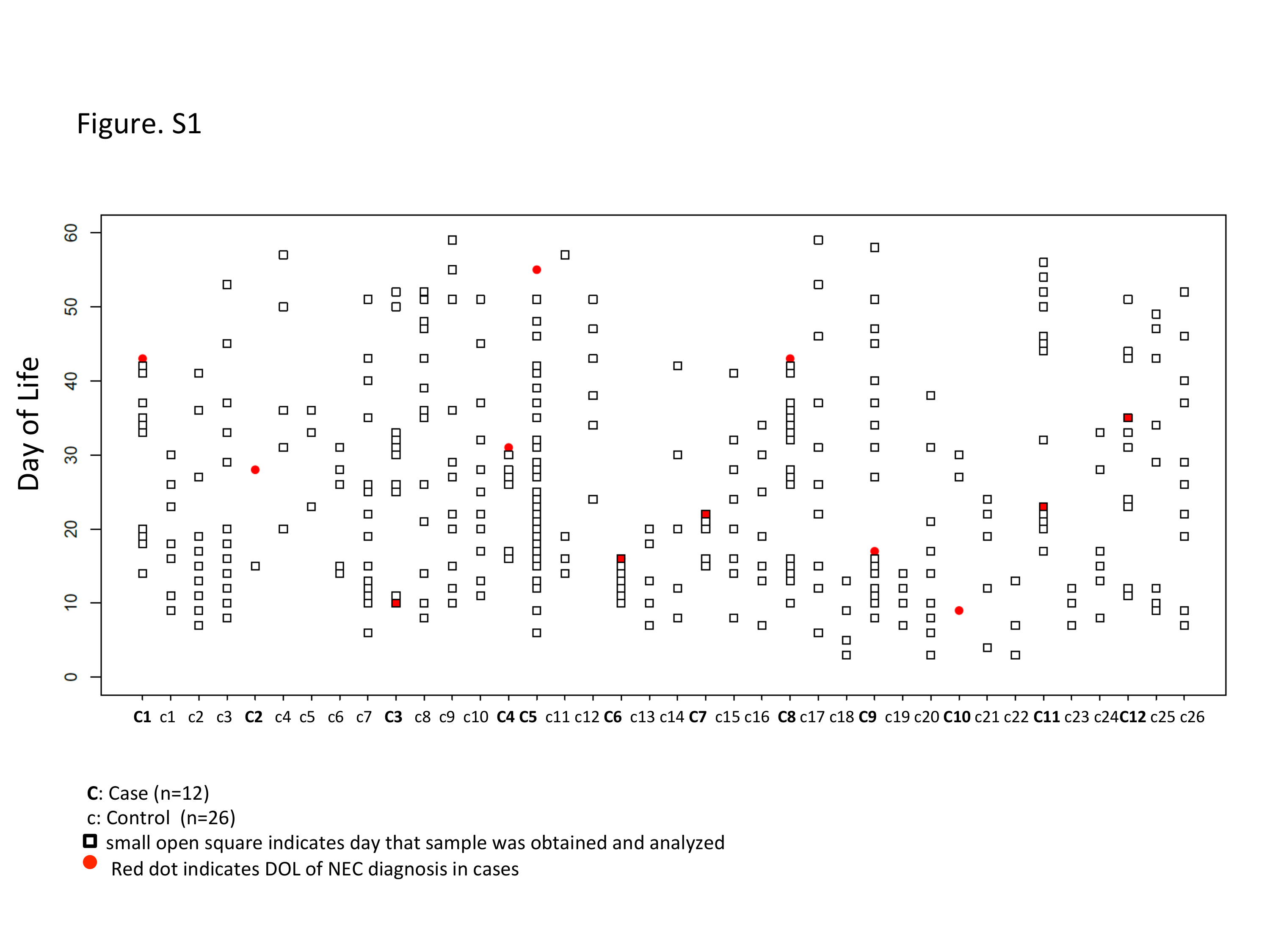

Supplement: S1 Fig — X-axis represents individual subject. Y-axis represents the day of life that a given sample is collected. (TIF) [file pone.0118632.s001.tif]

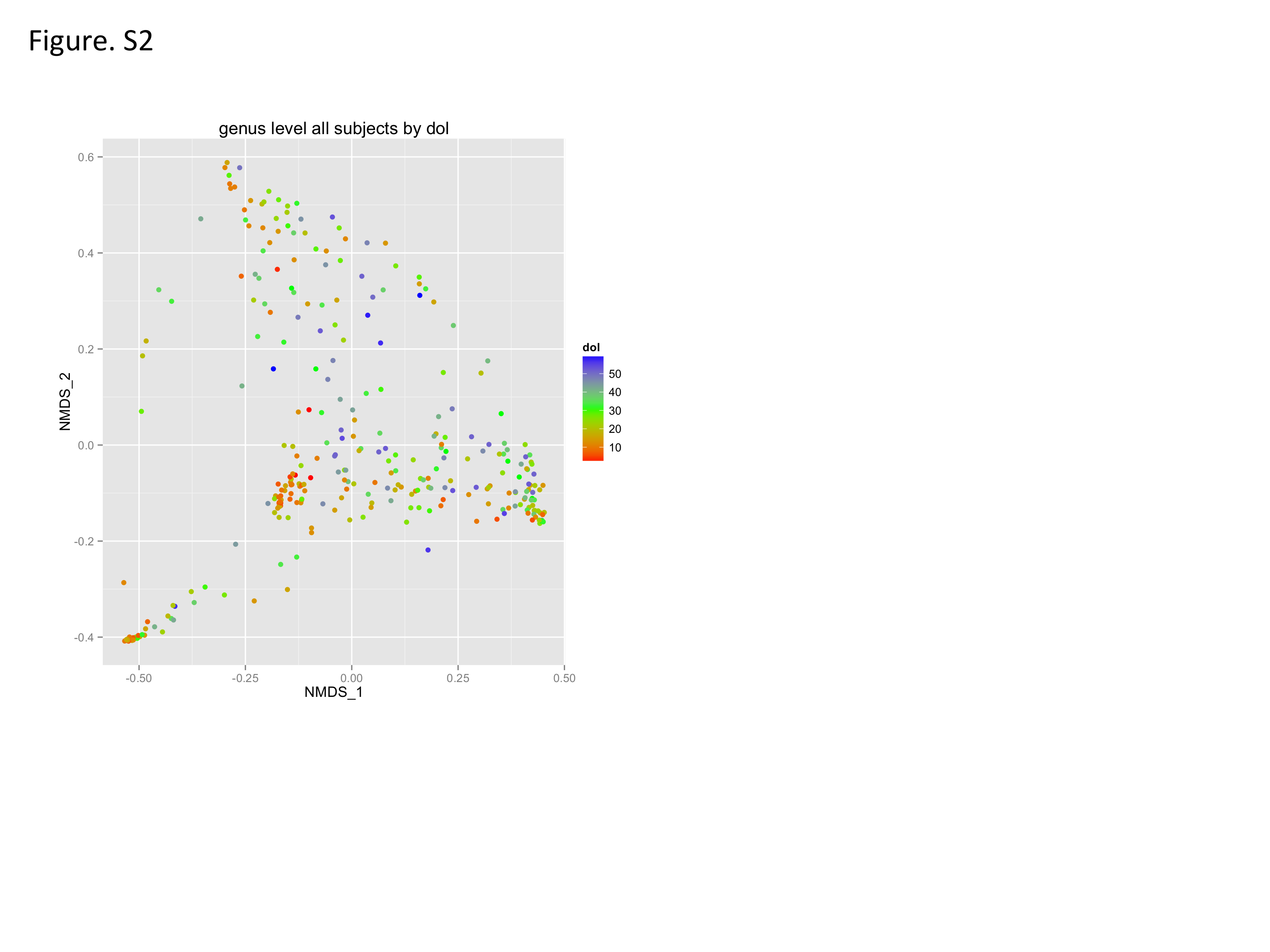

Supplement: S2 Fig — The gut microbiota variation at genus level over 0–60 days of life from all the samples is visualized by NMDS plot. The color gradient from red to blue represents the day of life of the babies. (TIF) [file pone.0118632.s002.tif]

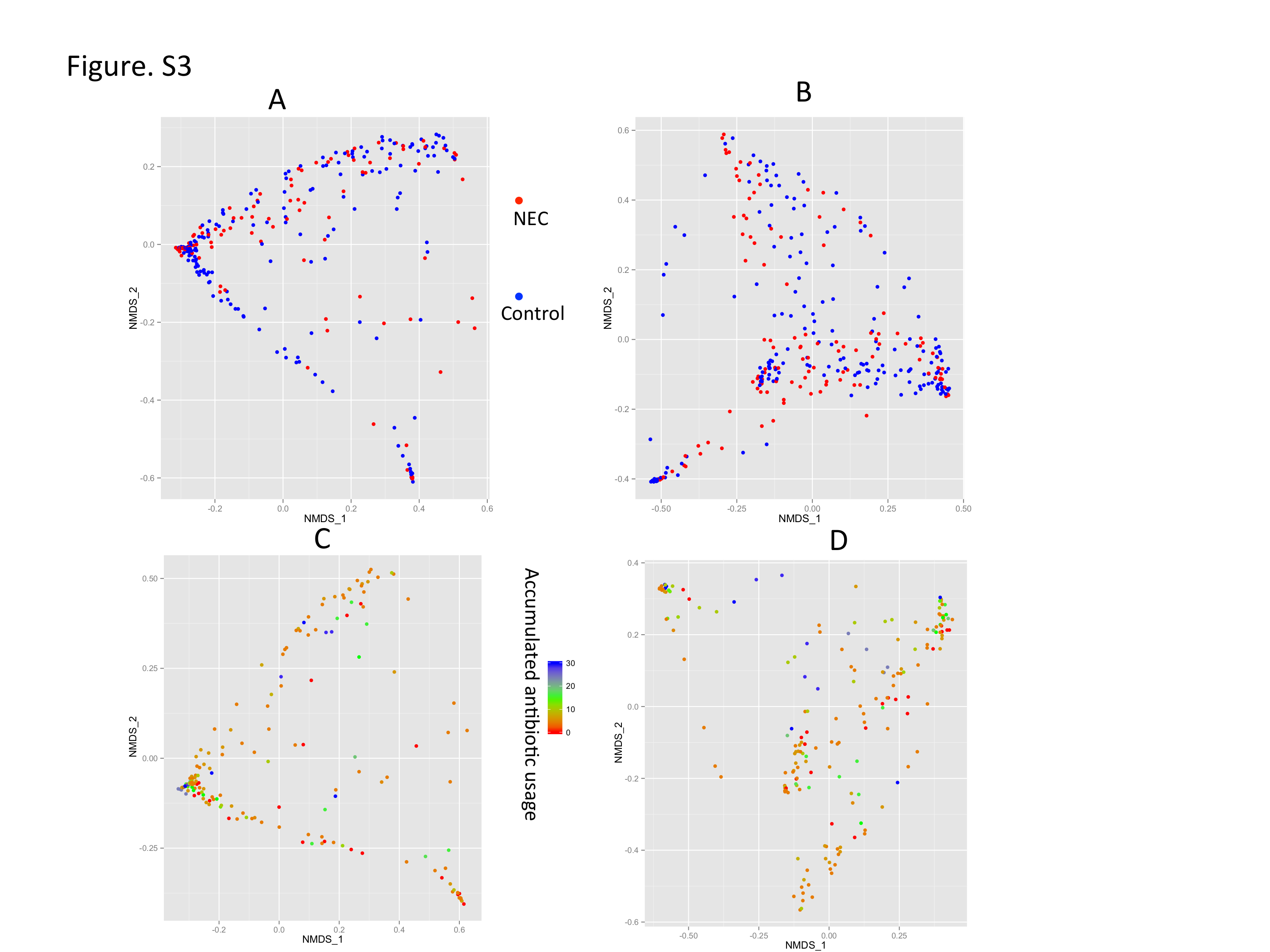

Supplement: S3 Fig — (A) NMDS plot at class level. Samples are colored by NEC or control groups (B) NMDS plot at genus level. Samples are colored by NEC or control groups (C) NMDS plot at class level. Samples are colored by the accumulated antibiotic usages. We assigned 1 for antibiotic usage and 0 without antibiotic usage. If multiple antibiotics were used at a given day, we summed up antibiotics to indicate the total antibiotic usage for the day. The color gradient shows the accumulated antibiotic usages overtime. (TIF) [file pone.0118632.s003.tif]

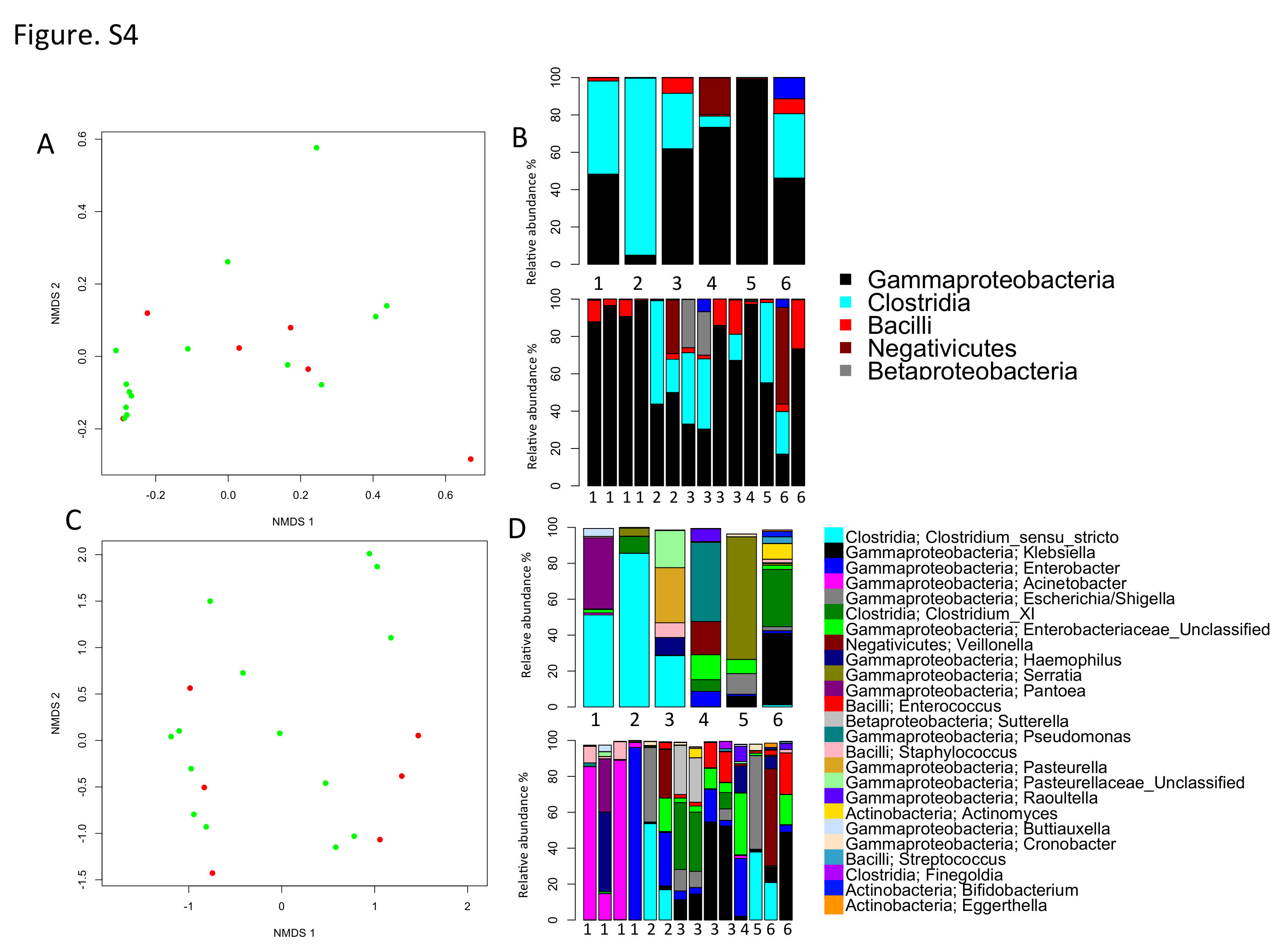

Supplement: S4 Fig — (A) NMDS plot of microbiota in NEC and controls at the class level at the day of NEC (B) The relative abundances of top five microbiota in 6 NEC samples (top) and their corresponding control samples (bottom) at class level. The NEC subjects are labeled as 1–6. 1–3: the early onset subjects; 4–6: the late onset subjects. Their corresponding control samples have the same label as the NEC cases. (C) NMDS plot of microbiota in NEC and controls at the genus level at the day of NEC diagnosis (D) The relative abundances of top 25 microbiota in 6 NEC samples (top) and their corresponding control samples (bottom) at genus level. The samples are labeled exactly as (B). The color scheme for each of the five taxonomical classes in S4B Fig. matches at least one of the color for the taxa within the same class. (TIF) [file pone.0118632.s004.tif]

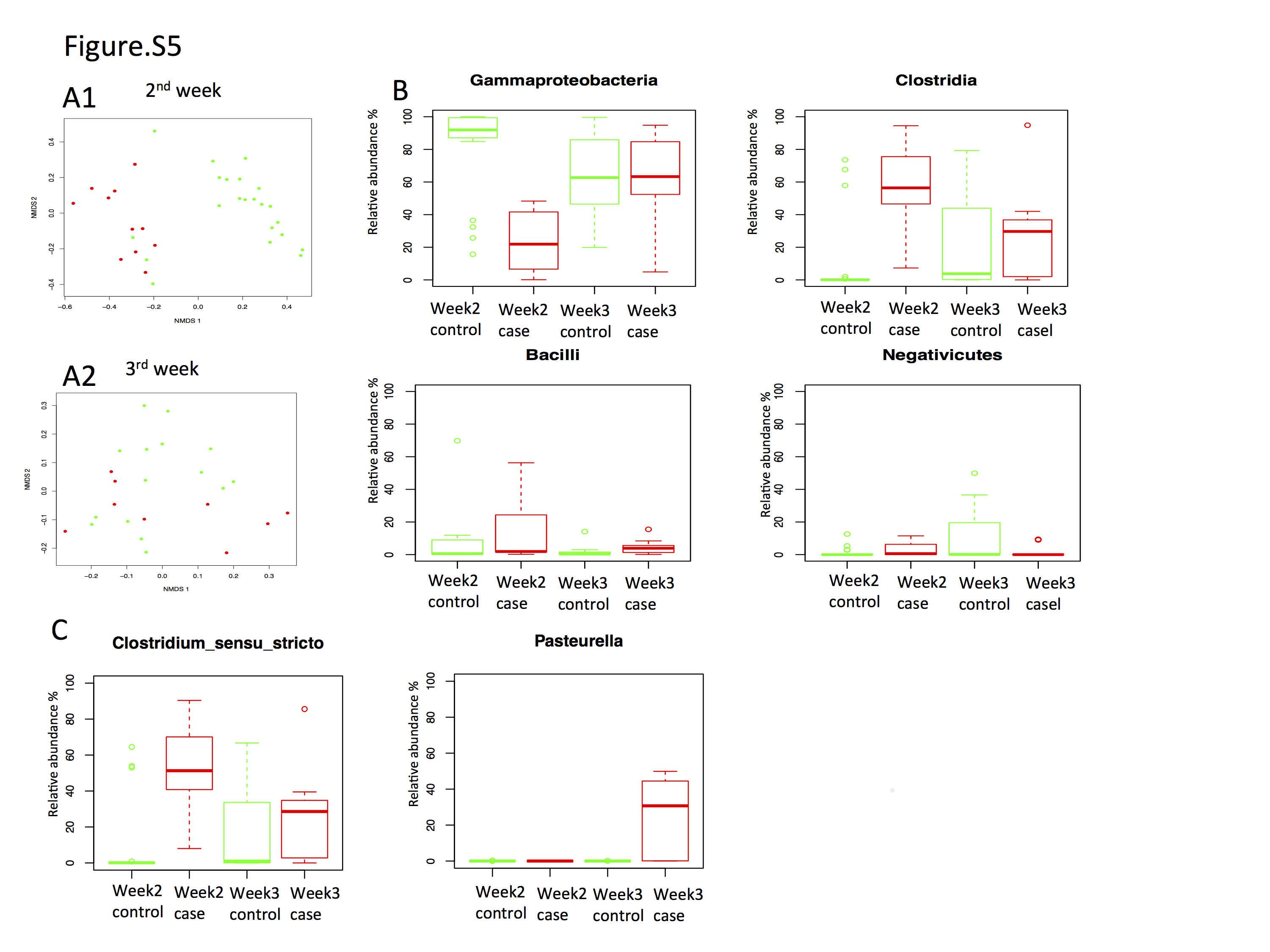

Supplement: S5 Fig — Each dot represents one sample.. All the control samples are coded by green, and all the NEC samples were colored with red in the NMDS plots (A1 and A2). The relative abundances of the four bacterial classes at the second and third week of life for NEC and controls samples (B). The relative abundances of two bacteria genera that are significantly different between second and third week (C). (TIF) [file pone.0118632.s005.tif]

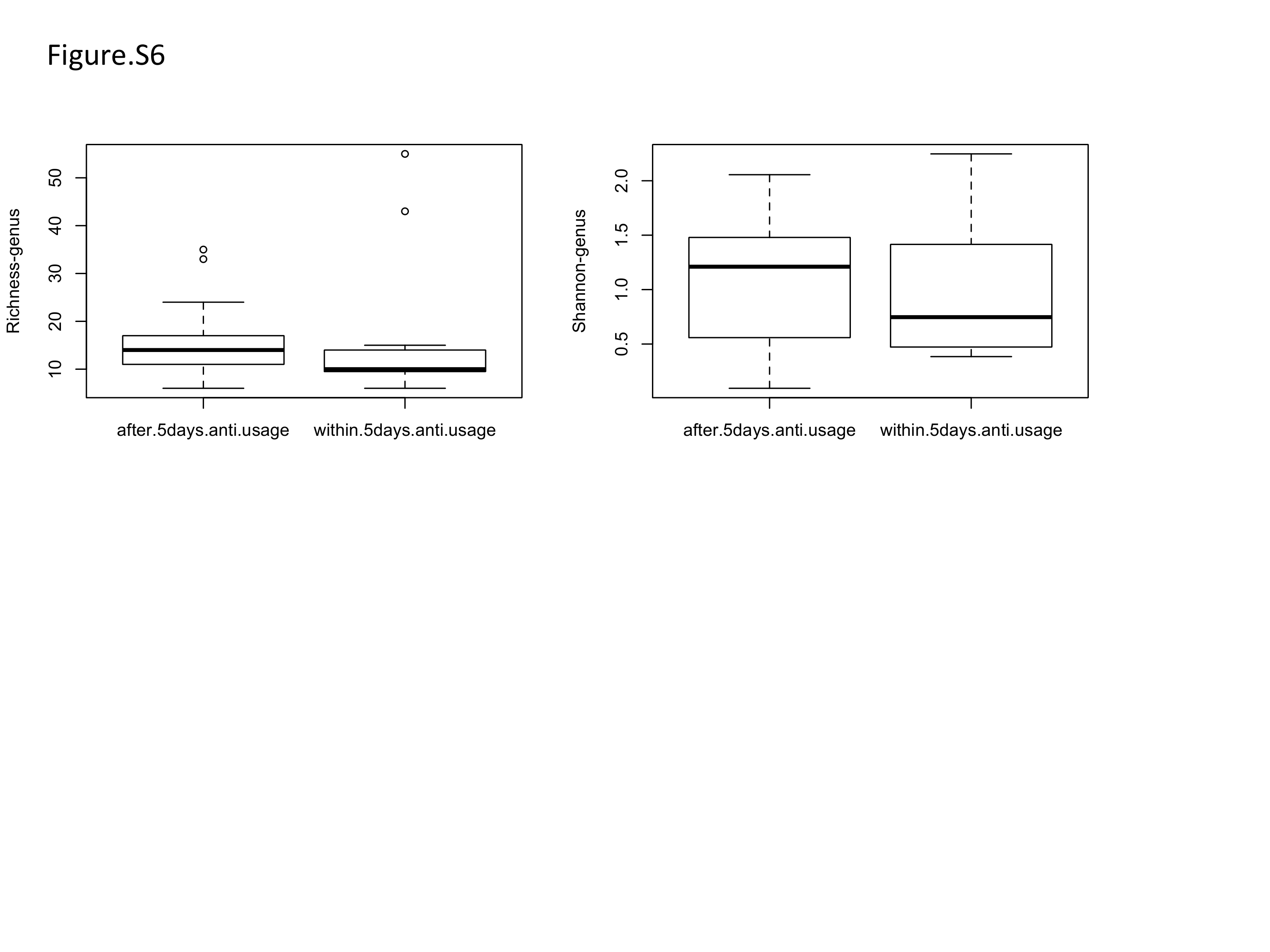

Supplement: S6 Fig — Richness (A) and Shannon diversity (B) are shown for samples treated with antibiotics within five days and samples without antibiotics for at least five days. The samples are from NEC control group from second week of the life. (TIF) [file pone.0118632.s006.tif]
