# Supplementary material for: Longitudinal Analysis of the Premature Infant Intestinal Microbiome Prior to Necrotizing Enterocolitis: A Case-Control Study
Source: PLoS One. 2015 Mar 5;10(3):e0118632. doi: 10.1371/journal.pone.0118632 (PMC4351051; doi:10.1371/journal.pone.0118632)
Supplement: S1 Table — (DOCX) [file pone.0118632.s007.docx]

Supplementary Table 1

| Early Onset NEC |  |  |  |
| --- | --- | --- | --- |
|  | Number of cases | Number of controls | p value (permanova) |
| week2 | 3 | 10 | 0.01 |
| week3 | 3 | 8 | 0.36 |
|  |  |  |  |
| Late Onset NEC |  |  |  |
| week2 | 4 | 12 | 0.06 |
| week3 | 5 | 9 | 0.05 |
| week4 | 5 | 8 | 0.05 |
| week5 | 5 | 9 | 0.12 |
| week6 | 3 | 7 | 0.34 |
